# Utilization of Health Care Services and Accessibility Challenges among Adults Aged 50+ before and after Austerity Measures across 27 European Countries: Secular Trends in the SHARE Study from 2004/05 to 2019/20

**DOI:** 10.3390/healthcare12090928

**Published:** 2024-04-30

**Authors:** Lena Borboudaki, Manolis Linardakis, Ioanna Tsiligianni, Anastas Philalithis

**Affiliations:** Department of Social Medicine, School of Medicine, University of Crete, 71500 Heraklion, Greece; linman@med.uoc.gr (M.L.); i.tsiligianni@uoc.gr (I.T.); philal@uoc.gr (A.P.)

**Keywords:** preventive health services, health care services utilization, lack of accessibility/availability, health inequalities, austerity, SHARE study

## Abstract

This study aimed to assess and compare the utilization of preventive and other health services and the cost or availability in different regions of Europe, before and during the economic crisis. The data used in the study were obtained from Wave 8 of the Survey of Health, Ageing and Retirement in Europe (2019/2020) and Wave 1 data (2004/5), with a sample size of 46,106 individuals aged ≥50 across 27 countries, adjusted to represent a population of N = 180,886,962. Composite scores were derived for preventive health services utilization (PHSU), health care services utilization (HCSU), and lack of accessibility/availability in health care services (LAAHCS). Southern countries had lower utilization of preventive services and higher utilization of other health services compared to northern countries, with a significant lack of convergence. Moreover, the utilization of preventive health services decreased, whereas the utilization of secondary care services increased during the austerity period. Southern European countries had a significantly higher prevalence of lack of accessibility. An increase in the frequency of lack of accessibility/availability in health care services was observed from 2004/5 to 2019/20. In conclusion, our findings suggest that health inequalities increase during crisis periods. Therefore, policy interventions could prioritize accessibility and expand health coverage and prevention services.

## 1. Introduction

Achieving universal access to health services, particularly for populations living in low- and middle-income countries, continues to be a major challenge of today and tomorrow, and efforts are needed to ensure timely and effective use of health facilities [1]. The need for increased health care utilization is associated with a growing elderly population, which poses as a potential burden on the financing of health care systems [2]. This burden further escalated due to the economic upheaval triggered by the 2008 global financial crisis. As a result, public revenues significantly decreased, while the need for publicly funded health care increased [3]. Both the aging population and comorbidity, which is particularly prevalent in the elderly population, inequalities in access, and economic pressures—magnified during the economic crisis—have led to the emergence of various new health care systems within the EU [4,5]. Complexity and fragmentation mark the term “access to the health system” [6]. Nevertheless, availability/accessibility, i.e., the ability to use health services when deemed necessary for the patient, stands out as a key indicator of access. Equally important is recognizing the deprivation of services when health needs cannot be met [7]. However, preventive and public health services are often not given priority, as in many countries the emphasis is often geared towards the development of secondary care services [8].

Social conditions are associated with health inequalities, as exemplified by the Diderichsen et al. model, which links vulnerability to systematic variations in living conditions due to socioeconomic differences [9]. Therefore, reconciling health inequalities, through a process of repeated observation of even subgroups within the population over time, access to reliable data and the interaction of determinants with health, in a system of monitoring of health inequalities, and the impact of health inequalities on the health of the population are crucial [10]. Furthermore, health policies can be successful if they are based on measurements that not only capture the problem, allowing informed decisions against health inequalities, but also facilitate the ongoing monitoring of the impact of the implemented measures [11,12]. The targeting of investments, guided by national monitoring of member states, is designed to improve health levels with a focus on equity within countries and to progress toward the Sustainable Development Goals (SDGs) [13].

In European countries, disparities in the health of the population have become evident in terms of improvement, particularly during the economic crisis. The majority of European countries have experienced an economic recession since 2007, resulting in increasing job losses and falling incomes, increasing national public debts. The result of these combined threats of falling output, rising unemployment, and escalating debts/deficits has been the subject of intense debate [14]. The effects of crises have been extensively studied [15,16]; however, it is important to investigate the differences between European countries in terms of the health services they used, pre- and post-crisis. For example, usage could have been reduced due to access barriers due to increased out-of-pocket payments and/or closure of public health facilities [17] or increased due to a deterioration in the health status of the population [16].

Over the past decade, European countries have shown varying trends in health indicators, health service utilization, and socioeconomic inequalities [12,18]. For example, European health systems that relied on out-of-pocket payments, with low levels of public spending on health, found themselves unprepared to cope with the 2008 financial crisis, consequently further weakening their resilience [3]. During the crisis period, private payments increased faster than public spending [19]. While public spending also increased, it did so at lower levels than before the crisis, negatively impacting progress towards universal health coverage. In southern European countries, the issue of health inequalities became a primary health policy issue in 2010, coinciding with the onset of the first effects of the 2008 economic crisis and austerity in southern countries [20,21,22]. For example, the period of austerity faced by Greece has had an impact on the size, structure, quality, and efficiency of health services [23,24]. Contrasting study results in Germany and Spain document that the economic crisis did not change the accessibility of the health care system in either country [25]. It is worth noting, however, that challenges in accessing health care services are not always related to the crisis, such as waiting lists being a long-standing problem [26].

Unlike northern European countries, the four southern countries still lack a coordinated and holistic response to social inequalities in health [27]. The increase in unmet health needs and economic hardship, particularly in countries with low levels of public health expenditure, led to longer waiting times, private burden due to increased user fees, and diminished access to publicly funded health services [21,28,29,30,31]. For example, in Greece, around 25% of the population lost social security coverage between 2011 and 2016 due to an increase in long-term unemployment and economic hardship that limited the ability to pay social security contributions [8,32]. However, the recent financial and economic crisis is likely to have affected health care services in two ways, either by increasing demand for some types of health services, or, due to reduced funding to the health care system at the same time as reduced incomes in the population, leading to a reduction in use due to a lack of access. In view of the above, ensuring access to high-quality health services in a period of crisis is particularly difficult for those who define health policies [26].

The pan-European economic shock, due to the crisis, creates a natural experiment in the policy of financial allocations for health care. Some countries such as Iceland, Greece, Ireland, Iceland, Ireland, the UK, Spain, and Slovenia have experienced large reductions in health spending, while others such as France and Switzerland have increased spending levels [14]. Factoring in the impact of the pandemic, health systems in Europe have been faced with the challenge of “dynamic preparedness”, that is, effective management of COVID-19 morbidity, but also unhindered access to essential health services for non-COVID-19 patients [33,34].

In addition, several studies have confirmed that the implementation of structural adjustment policies against the economic crisis in southern European countries has had the side effect of increasing social inequalities [35,36]. Therefore, it is well documented that health inequalities have worsened in European countries during the economic crisis of the 2010s [37]. However, there is a lack of/controversial empirical evidence on the current status of health inequalities in Europe [38]. Consequently, the aim of this study was to examine the utilization and accessibility of health services for adults aged 50+ years in 27 European countries before and after the onset of the economic crisis. Furthermore, the aim was to compare the utilization of preventive and other health services, as well as the lack of accessibility to health services in northern, central, and southern European countries by examining health service utilization before and after the economic crisis (secular trends, 2004/5–2019/20).

## 2. Subjects and Methodology

### 2.1. Study Population and Sampling

Participant data from the 8th wave of the “Survey of Health, Ageing and Retirement in Europe—SHARE” (“Survey of Health, Ageing and Retirement in Europe—SHARE”, http://www.share-project.org/home0.html, accessed on 14 March 2024) were used [39,40,41]. Data were collected in 2019/20 from 26 European countries: Austria, Belgium, Bulgaria, Croatia, Cyprus, Czech Republic, Denmark, Estonia, Finland, France, Germany, Greece, Hungary, Latvia, Lithuania, Luxembourg, Malta, Netherlands, Poland, Romania, Slovakia, Slovenia, Spain, Italy, Sweden, Switzerland, and Israel [41,42]. The research was organized and coordinated by the Research Institute of Mannheim, Germany, and it is a collaborative effort of interdisciplinary, national, and transnational working groups (the Greek group consists of members from the Universities of Panteion, Piraeus, and Crete). In Wave 8, based on the available data (Release data 8.0.0/10.02.2022), 46,733 individuals aged 32–104 years (or 46,547 aged 50+ years) participated. The total study sample consisted of representative, stratified, composite samples of adults, selected proportionally in each country through probability sampling. Since the beginning of the study (Wave 1, 2004/5), the target population has been households and, by extension, their members, where at least one member was 50+ years old [39,40]. The current analysis sample amounts to 46,106 persons 50+ years old from 27 countries which is corresponded to a target population of N = 180,886,962 persons based on selection weights.

### 2.2. Research Tool

The data were collected from the Survey of Health, Aging, and Retirement in Europe (SHARE) project, and tools were from SHARE (https://share-eric.eu, accessed on 14 March 2024). Data collection was conducted through face-to-face interviews via computer using the CAPI questionnaire, which consists of 31 modules. These modules covered various aspects such as respondents’ (or household members or their proxies) demographics, social networks, physical and mental health, employment and retirement, cognitive function, etc., or measures such as handgrip strength or walking speed. In some sections, pre-selected cards helped with understanding and answering the questions directly and reliably [43].

#### 2.2.1. Preventive Health Services Utilization Score (PHSUs)

Preventive health services utilization (PHSU) was assessed using a composite score using 7 questions. The questions assessed whether participants had the following: (1) supplementary insurance; (2) flu vaccination; (3) eye examination; (4) a mammogram; (5) a colon cancer screening; (6) a planned hospitalization; (7) polypharmacy; all questions were coded as a binary variable (0 = no/never; 1 = yes/at some time/every visit), and a composite score (range = 0–12) was calculated by summing responses [44]. The score was subsequently rescaled to 0–100, with higher values indicating higher preventive health services utilization [44,45].

#### 2.2.2. Health Care Services Utilization Score (HCSUs)

Health care services utilization (HCSU) was assessed by a composite score using 15 questions. The questions assessed how often participants had undergone the following: (1) times talked to medical doctor/nurse; (2) contact with general practitioners; (3) contact with specialists; (4) been a patient in hospital; (5) been a patient in a nursing home; (6–9) received home care; (10) seen a dentist/dental hygienist; (11) total nights stayed in hospital; (12) stayed overnight in hospital; (13) paid for nursing care; (14) hours received professional nursing care; (15) hours received paid domestic help.

All answers to questions were coded as a binary variable (0 = no/never; 1 = yes/at some time/every visit), and a composite score (range = 0–16) was computed by summing responses. Subsequently, the score was rescaled to 0–100, with higher values indicating higher health care services utilization. The HCSU score is a new index that was used for the first time in 2020 [45] and is used with modifications to certain variables in this article, in order to achieve comparability between the 1st wave (2004/5) and the 8th wave (2019/20).

#### 2.2.3. Lack of Accessibility/Availability in Health Care Services (LAAHCS)

Lack of accessibility/availability in health care services (LAAHCS) was assessed by a composite score using 16 questions. The questions assessed how often participants had forgone care due to the cost of the following: (1) GP; (2) specialist physician; (3) drugs care; (4) dental care; (5) optical care; (6) home care; (7) paid home help; (8) other care. Furthermore, they assessed how often participants had forgone care due to unavailability of the following: (9) GP; (10) specialist physician; (11) drugs care; (12) dental care; (13) optical care; (14) home care; (15) paid home help; (16) other care. Their frequency of use is low, as when they are aggregated, a composite score is obtained where 87% have zero use (lack or no accessibility to the specific 16 services), and it was divided into score frequency as follows: 0 (zero score) or no lack of accessibility (no lack of accessibility), 1 to 24 or partial lack (partial lack), and 25+ or high lack of accessibility (high).

#### 2.2.4. Socioeconomic Characteristics

The social and demographic variables included in the study were gender, age, educational level, and living conditions. Age was categorized into four groups (50–59, 60–69, 70–79, and 80+ years), while living conditions included two categories: “living alone” and “living with a partner/spouse”. Years of education were calculated on the basis of the total study time at different levels of education as defined by national education systems [46]. Economic status was recorded as the gross household income in the previous year. Reflecting transnational differences in household income, the quadrants were calculated and used by country [44,45]. The countries were grouped by region into northern Europe (Denmark, Estonia, Finland, Latvia, Lithuania, and Sweden), central Europe (Austria, Belgium, Czech Republic, France, Germany, Hungary, Luxembourg, the Netherlands, Switzerland, Poland, Romania, Slovakia, and Slovenia), southern Europe (Bulgaria, Croatia, Cyprus, Greece, Italy, Malta, and Spain), and Israel.

### 2.3. Statistical Analysis

Data were analyzed using the SPSS software package (IBM SPSS Statistics for Windows, version 25.0., IBM Corp., Armonk, NY, USA). Relative and absolute distributions of the descriptive characteristics of participants were estimated as weights were applied according to the complex multistage stratification sampling design of the study, accounting for non-responses. Using weights, the results were extrapolated to the actual estimated reference population of the countries [40]. The prevalence and comparative corresponding 95% confidence intervals (95% CIs) of the PHSU, HCSU, and LAAHCS components were estimated. Across the 27 countries, the PHSUs, HCSUs, and LAAHCSs were also illustrated as a spider diagram. Score frequency of LAAHCS in relation to characteristics of participants was assessed based on χ^2^ (chi-square) method. In addition, the mean PHSUs and HCSUs were assessed and compared between European regions or according to score frequency of LAAHCS, using analysis of covariance (ANCOVA) that also estimated the corresponding 95% CIs for comparative reasons. Estimations were based on complex samples analysis using as covariates (potential confounders) the basic characteristics of participants such as gender, age distribution (years), education status (years), family status, occupation, and chronic conditions or diseases.

## 3. Results

From n = 46,106 Europeans aged 50+ who participated in the current study, 51.5% were from the central European region (Table 1), 57.4% of all were women, 69.0% were seniors (60–79 years) and 20.8% were elderly (80+), and average participant age and years spent in education were 71.3 and 11.8 years, respectively. Concerning living, occupation, and health status, 12.8% were found to be unmarried/divorced/widow, 79.1% were unemployed/retired/housemaker, 74.1% were with one or more at least one chronic conditions or 24.2% were also reported as having three or more chronic conditions.

Table 2 shows the prevalence of the seven components of PHSUs, where, among others, 32.4% reported having received an influenza vaccine in the last year, 50.5% reported having been examined in the last 2 years by an ophthalmologist or optometrist, 22.0% with polypharmacy, while in diagnostic examinations, 25.5% and 28.6% of the participants underwent mammography and sigmoidoscopy/colonoscopy, respectively.

Similarly, Table 3 shows the prevalence of 15 components of HCSUs, where, among others, 9.9% reported not being seen by a GP in the last year, 3.3% reported 13+ GP visits per year, and 15.8% reported no visits. Additionally, 37.7% were not seen by a specialist in the last year, and only 2.8% had 13+ visits. In total, 5.4% were hospitalized in the last 12 months 2+ times, 0.4% in the last year had a stay in a care home for one night. Furthermore, 2.4% reported receiving professional home care (nursing or personal care or weeks receiving nursing care), and 6.9% reported receiving professional home care (domestic work or weeks receiving help from paid professionals). Only 2.2% received care from private providers, and 55.3% reported that in the past twelve months they had visited a dentist/dental hygienist. Furthermore, 14.9% reported that they were hospitalized at least one night in a medical, surgical, psychiatric, or any other specialized department.

Also, Table 4 shows the prevalence of 16 components of LAAHCS, wherein inability due to cost was reported at a higher frequency, only 4.5% had received dental care, and 2.5% had visited a specialist physician. Similarly, inability due to unavailability was reported with a higher frequency: 2.9% could not visit a specialist physician, only 1.7% had received dental care, and only 1.2% had visited an optometrist.

Table 5 shows the extracted score levels of PHSU and HCSU between European regions. As the scores ranged from 0 to 100 and higher levels, indicating a greater use of health services, participants from northern European countries in relation to participants from southern Countries were found to have significantly higher mean levels of PHSUs (26.2 vs. 22.2, *p* < 0.05). In contrast, participants from northern European countries in relation to participants from southern and central countries were found to have significantly lower mean levels of HCSUs (13.4 vs. 15.8 and 17.2, respectively, *p* < 0.05). Presenting the difference in the use of health services, in northern countries the PHSUs is greater than the HCSUs by 12.8 points in contrast to the south, which was lower by 6.4 points (*p* < 0.05). In relation to 27 countries (results not shown in table/figure), participants from Belgium were also found with the highest PHSUs and HCSUs (32.4 and 21.0, respectively). Participants with the lowest PHSUs were from Bulgaria (7.9), and those with the lowest HCSUs were from Malta (11.0).

**Figure 1:**
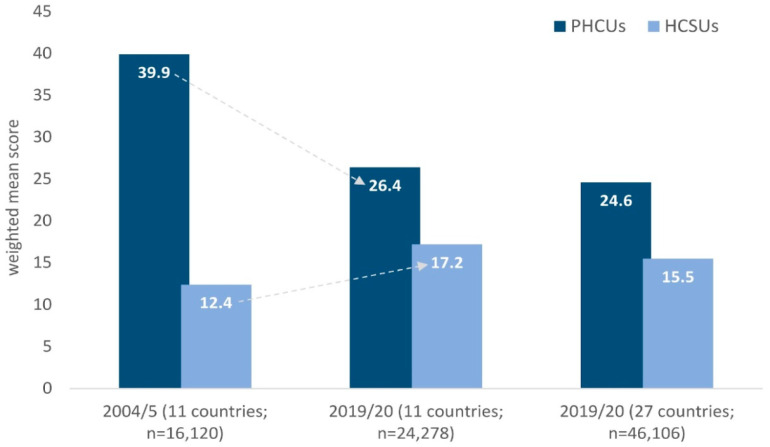
Score levels of preventive health services utilization (PHSU) and health care services utilization (HCSU) in the samples of Waves 1 (2004/5) and 8 (2019/20) of European adults, aged 50+ years. PHSUs: preventive health services utilization score; HCSUs: health care services utilization score. Data of “2004/5 (11 countries; n = 16,120)” were drawn from Borboudaki and colleagues (2021) [45].

**Figure 2:**
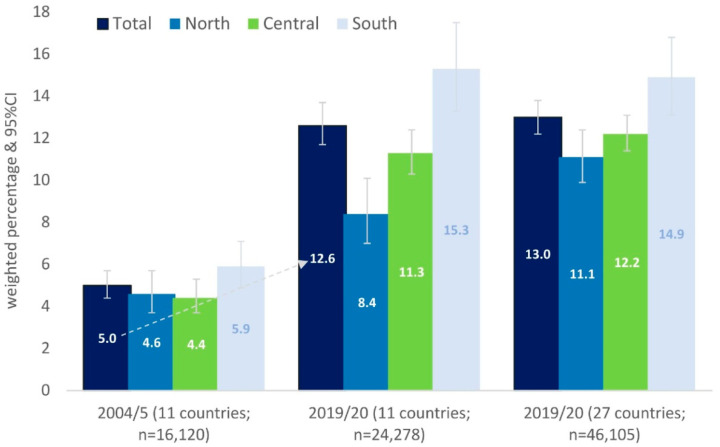
Frequency of lack of accessibility/availability in health care services (LAAHCS) in the samples of Waves 1 (2004/5) and 8 (2019/20) of European adults, aged 50+ years, between European regions.

Figure 1, despite methodological differences, compares the levels of PHSUs and HCSUs at 15 years, from Wave 1 (2004/5) to Wave 8 (2019/20). There is a significant decrease in 11 countries from 2004/5 to 2019/20 in PHCUs from 39.9 (95% CI 39.4, 40.4) to 26.4 (95% CI 25.9, 26.8) and a significant increase in HCSUs from 12.4 (95% CI 12.2, 12.7) to 17.2 (95% CI 16.9, 17.5). As for the total number of participants (27 countries), PHCU scores seem to decrease from 2004/5 to 2019/20 and HCSU scores increase. However, for LAAHCS (results not shown in table/figure), southern countries were found with a significantly higher frequency of scores of 25+ (high lack of accessibility) (1.3%; 95% CI 1.0, 1.6) compared to northern countries (0.4%; 95% CI 0.3–0.5) or generally with a higher frequency of lack of accessibility. As shown in Figure 2 and corresponding to Figure 1, a significant increase is observed in LAAHCS in the 11 countries from 2004/5 (5.0%; 95% CI 4.4, 5.7) to 2019/20 (12.6%; 95% CI 11.7, 13.7). In fact, in the 11 countries, in 2004/5 there seems to be no north–south difference (4.6% vs. 5.9%), while in 2019/20 it almost doubles (8.4% vs. 15.3%).

In Table S1 (Supplementary Materials), where LAAHCS in relation to characteristics of 46,105 Europeans adults is related, it is found that there is a significantly higher prevalence of lack of accessibility (score 25+) for women compared to men (1.1% vs. 0.9%, *p* < 0. 001), having family status as unmarried/divorced/widow versus those who are married/living with a partner (2.3% vs. 0.8%, *p* < 0.001), having occupation status as unemployed/retired/housemaker versus those who are employed (1.2% vs. 0.6%, *p* = 0.017), or having 3+ chronic conditions versus those who do not (1.6% vs. 0.6%, *p* < 0.001).

Finally, Table 6, which compares the score levels of PHSU and HCSU between the three LAAHCS categories, shows that participants with higher frequency scores (25+ or high accessibility deficiency) versus those with no accessibility deficiency (0 score) were found to have significantly lower PHSU scores (18.52 vs. 24.71, *p* < 0.001). Meanwhile, participants with a higher frequency of scores (25+ or high lack of accessibility) versus those with a partial lack of accessibility (partial lack) were found with significantly lower HCSU scores (14.61 vs. 16.64, *p* < 0.001). At the same time, they are estimated to have significantly less discrepancy between the two types of services, as the gap is less than double in prevention use between the categories; thus, a higher lack of accessibility determines lower use of both types of health service use.

## 4. Discussion

Based on the above results, interesting data emerged regarding the use of preventive health services and secondary health services, as well as differences in accessibility, before and after the financial crisis. In our survey, we observed a significant decrease in the utilization of preventive health services across 11 countries from 2004/5 to 2019/20 and an increase in the use of secondary health services. A similar pattern holds for all 27 countries in Wave 8. Also, the prevalence of the PHSUs, preventive medicine services variables, recorded low levels. We also recorded a significant change in the incidence of lack of accessibility/availability in health care services (LAAHCS) from 2004/5 to 2019/20. In 2004/5, there did not seem to be a north–south difference, while in 2019/20 it is significant. Central European countries were found to have significantly higher mean PHSU and HCSU scores than their counterparts elsewhere in Europe (*p* < 0.05), as well as a larger gap between the two scores. The southern countries had lower preventive use and higher service use than the northern countries (*p* < 0.05) and significantly less convergence between them. Additionally, participants with a higher prevalence of a lack of accessibility compared to those without a lack had significantly lower prevention and health service use scores.

Research indicates that during the crisis, health care deprivation increased in Europe, widening social inequalities, with the exception of countries with relatively equal income distribution, which managed to protect their populations, especially vulnerable groups [46]. According to the ATLAS survey, wealth had a strong effect on baseline scores, with participants with lower levels of education and wealth generally having lower healthy aging scores [47]. The results of the study also support the hypothesis that higher-income patients tend to wait less for access to primary care in four high-income countries: Canada, Germany, Germany, Norway, and Sweden [48].

In addition, in research studies, there was a correlation between austerity and an increase in the incidence of serious mental illness (SMI). Variations were recorded in the incidence of SMI, due to deprivation associated with changing socioeconomic status. The above are basic elements for implementing targeted interventions and strengthening the social welfare system [49].

Our findings, aligned with those of other studies, confirm that during the years of the crisis, when affordability declined, we had a decrease in the use of preventive health services, which is likely to be correlated with the increase in the use of secondary care recorded in our results.

In our study, the prevalence of the PHSUs, preventive medicine services variables, recorded low levels. For example, 25.5% and 28.6% of participants underwent mammography and sigmoidoscopy/colonoscopy, respectively. Only 32.4% reported vaccination for influenza. The low rates of utilization of PHC may be due to the fact that the sources of expenditure are fragmented and rely mainly on private payments [50]. The priority given to primary health care and the implementation of screening in allocation of public expenditure vary considerably between European countries.

The decline was higher and more prolonged in Cyprus, Croatia, Greece, Ireland, Italy, Spain, Latvia, Portugal, and Slovenia. It is underlined that Cyprus, Greece, Ireland, Portugal, and Spain received financial support from the European Union, in order to carry out reforms, applying health austerity measures with the aim of long-term development [51]. Moreover, Italy and Estonia implemented health austerity measures, albeit at different levels, even though the Troika did not impose austerity on these countries [52]. On the other hand, Greece is a country with low rates of screening and utilization of PHC services, because care is highly fragmented, with several different public and private providers involved, without coordination between them [53]. In 2022, the institution of the family doctor was adopted in Greece, but a significant coordination effort is still needed, since, as documented by the health committee of Hanson et al. not only for Greece but more broadly, public funding for PHC is inadequate, access is unequal, and patients are charged out of pocket for using services [48].

Research also indicates that as the severity of illness and disability increases in the period preceding death, older individuals may prefer professional care to alleviate the burden on family members [54]. However, in our study this was not confirmed, as 97.8% reported receiving no professional or paid personal care assistance at home (in the last 12 months), and only 6.6% reported receiving more than one hour of home help. However, the accessibility to end-of-life health services is a global priority, including palliative care and other social care services [55,56]. In our study, participants with a higher prevalence of lack of accessibility compared to those with no lack (0 score) have significantly lower prevention and health service use scores (*p*-trend < 0.05). Lack of access to prevention services is inversely proportional to morbidity and therefore to the need for hospitalization and high health care costs. Scientific research shows that the likelihood of hospitalization decreases as the use of preventive health services increases, while reducing health care costs [57,58]. Researchers have also shown that comorbidity increases overall health care costs [59,60,61]. Comorbidity is associated with both age and a reduced use of preventive health services aimed at disease prevention. Therefore, political will and social consensus are necessary to obtain integrated, continuous, and effective PHC [62] and implement treatment guidelines, leading to improved quality and rationalization of health care costs. The fact that population aging affects the formulation of health care policies and the prediction that in 2070, 30% of people living in European countries will be aged 65 years and people aged 80 years and over will more than double, reaching 13% [63], creates the need for immediate implementation of health policies aimed at increasing the provision of PHC services and reducing vulnerability. The impact of aging on health systems and their sustainability [64,65] is significant, leading to an ever-increasing demand for health services [66]. Older people suffer from comorbidity, consuming more health resources than the general population [67], which explains 74% of health resource consumption [68]. Universal coverage is linked to appropriate policies aimed at reaching vulnerable groups [69] (Hashiguchi TCO., Llena-Nozal A., 2020).

Our results are confirmed by other studies that have documented, during the years of the economic crisis, cuts in public spending on health care, increases in user fees and decreases in disposable income, decreases in health care consumption due to increases in health care spending, and decreases in income [70,71,72,73,74]. A similar study showed that over the period 2008–2015, the proportion of the population with severe material deprivation escalated from 11.2% to 22.2%, while the proportion of the population without access to health services due to financial hardship increased from 4.2% to 10.9% over the same period [74]. Similarly, another study in the EU reported that during the crisis period, the population had difficulty accessing medical needs. Even in countries where the majority of the population do not report an access problem due to distance, cost, or waiting time, over a third experience at least one of these problems: 35% in Sweden, 41% in the Netherlands, 43% in Finland, 44% in Spain, and 45% in Denmark. In addition, population aging is putting pressure on health systems, as well as cross-border flows of patients [26].

According to the results of the present survey, a significant difference between southern and northern countries was recorded, with southern European countries showing a significantly higher prevalence of lack of accessibility. According to Kyriopoulos et al., austerity measures during the financial crisis have been associated with hospital mergers and a reduction in health workers, with a negative impact on accessibility and availability [75]. Our study recorded a significant change in the frequency of lack of accessibility/availability in health care services (LAAHCS). In fact, in the 11 countries, in 2004/5 there did not seem to be a north–south difference, while in 2019/20 it is significant. In our study, southern countries have lower preventive use (22.2) and higher service use (15.8) than northern countries (*p* < 0.05) and have significantly less convergence between them (Δ = 6.4) (prevention with use is very close, unlike in northern countries). Instead of the development of PHC becoming an opportunity to deal with the effects of the economic crisis, it became a field for saving resources. In contrast, the health systems of Germany, the UK, Sweden, and the Netherlands have prioritized preventive health services [76]. Wensing et al. emphasized the positive effects of robust PHC on both health and the economy. During the COVID-19 pandemic, the need to strengthen primary care services was great, as reduced access to hospitals led to a gap between supply and demand for health services [77,78]. However, the impact on health is not only determined by the economic crisis but also by the properties of the welfare state [79], because in addition to the crisis, political decisions also determine the impact of the crisis on health [80,81]. In particular, Iceland, which did not implement austerity policies, did not record adverse health outcomes despite economic collapse and stagnation, unlike Greece, Spain, and Portugal, which implemented harsh austerity policies [81,82]. The welfare state, despite the current economic situation, still retains its resilience and importance for all European countries [83].

In our results, participants from central European countries were found to have significantly higher mean PHSU and HCSU scores than their counterparts elsewhere in Europe (*p* < 0.05), as well as a larger gap between the two scores. Overall, the respective mean PHSU and HCSU scores were 39.9 and 12.4. This is also documented in other studies, as European countries experienced significant negative impacts due to the European economic and financial crisis in previous years [82,84,85]. Greece, Spain, and Portugal have necessarily experienced harsh fiscal austerity [85,86,87]. Each country responded differently and adapted to the austerity measures, but the effects of the financial crisis on the general population are strikingly similar [85,88,89]. It should be noted that within the EU, some countries (e.g., the Czech Republic, Estonia, Italy, Lithuania, Slovakia) were better prepared than others because of the fiscal measures taken before the crisis [90]. In other countries, health budgets were protected (Belgium, Denmark) [91].

Our results reconfirm that health inequalities increase during periods of crisis, as suggested by other studies [92,93,94,95,96]. One study recorded a sharp increase in inequalities in 2010, coinciding with austerity measures in EU countries having an impact on socioeconomic inequalities [92]. Heggebo and Dahl documented that countries of the former “eastern bloc” (Estonia, Latvia, Lithuania, and Hungary) together with Portugal tend to report the highest prevalence of limiting long-term sickness (LLSI). In contrast, Nordic countries (excluding Finland) and Benelux countries report comparatively low levels of LLSI [97]. Chauvel and Leist also found income inequalities across countries and income-related health gradients, with low-income status being associated with health problems in vulnerable individuals [98]. In a survey conducted by Kyriopoulos et al., it was found that 25% of chronically ill patients faced geographical barriers, while 63.5% and 58.5% of them faced financial hardship and waiting list delays, respectively. Inequalities can exacerbate the health status of chronic patients, which brings about adverse effects on health care costs [75]. More importantly, another study suggested that austerity measures have been linked to a decrease in access to various categories, including an increase in unmet needs, affordability, suitability, and availability and accommodation, with vulnerable populations, such as the elderly, having been hit the hardest by austerity measures in terms of access to health services [52]. One study found that in Spain and Germany, the use of services decreased between 2009 and 2017. This decrease coincided with the period of austerity in Spain. However, no socioeconomic differences were found in the use of health services [99]. Restrictions on health spending during the Great Recession caused in many European countries reduced access to the health system [25,82] and an increase in self-reported unmet medical needs [100]. The mortality rates observed in the years 2011–2016 were significantly higher than expected in the age groups 0–4 and 65–74, with an increase in mortality from various medically amenable conditions [101]. Furthermore, and in light of unforeseen war scenarios in Europe and globally, health equity takes on a central role, and it is deemed necessary to ensure social justice by reducing inequalities in the care of young, underserved populations and refugees [102]. Measuring, recording, and monitoring health inequalities is the solution to ensure health equity [103].

The emergence of the new coronavirus that caused the COVID-19 pandemic led to a global public health crisis, accompanied by a simultaneous increase in demand for health and medical services [104]. Health systems around the globe were reorganized to adapt and deal with uncertainty, leaving in some cases concerns about equity [105,106]. Due to the health crisis, many non-emergency medical treatments and scheduled medical appointments were postponed or cancelled, with a huge impact on non-acute health conditions, especially in vulnerable populations such as the elderly [106]. Therefore, the COVID-19 pandemic further highlighted the necessity of having a well-functioning PHC, establishing it as a key component of health systems with high efficiency and universal health coverage. Therefore, a successful reorientation towards PHC with smart policies and long-term commitment, taking into account the social and economic context, is therefore required [50]. The WHO reported on the impact of the pandemic not only on the physical but also on the mental health of the population [107,108], with a significant decrease in mental health services [99,104], however, as mainly telephone support lines were used in many countries, a service provided that, although it contributed significantly, could not alone address the significant mental health footprint of incarceration and the burden on the population’s mental health due to the pandemic. Early evidence suggests that risk factors associated with the pandemic and COVID-19 lockdown are leading to an exacerbation of mental health symptoms [109,110,111]. During the pandemic, several social and demographic characteristics significantly determined patterns of health service use. For example, a study showed that people over 61 years of age with comorbidity reported higher rates of health service use due to poor health status, whereas people of the same age with better health status reported a key reason for increasing health care utilization rates was COVID-19 disease [112]. Both during and prior to the pandemic, significant barriers to accessing mental health services were documented, including cost, lack of insurance coverage and acceptance, and long waiting times [111,113,114]. Research has shown that populations of low socioeconomic status and racial/ethnic minorities are more vulnerable in times of crisis [115]. The COVID-19 pandemic has led to health care models that reduce face-to-face contact between clinicians and patients. Telemedicine has made significant inroads into primary care delivery, although its effectiveness remains unclear. However, it enables the provision of timely, immediate, and lower-cost interventions at a distance, while improving access to health care [113]. In Europe, it has been the preferred mode of primary care delivery, significantly reducing face-to-face visits unless deemed necessary by the treating physician [113]. The use of health services during the health crisis, whether related to COVID-19 or not, showed a downward trend as the volume of hospitalized patients decreased [104]. A study in the Netherlands documented that oncology care decreased and cancer screening programs were neglected [116]. In a large study in China, a significant number of the population made use of mainly primary health care, particularly in response to the Omicron mutation [117], as symptomatology was mild, and cases could be treated with medication without the need for hospitalization [104]. The pandemic also highlighted the need to keep pharmacies open and accessible to the public for the smooth supply of medicines to the population. Also important is the existence of online pharmacies as an option to ensure smooth supply of medicines to the population during crises [118]. Conversely, the pandemic modified the way rehabilitation care is delivered to patients, especially in the geriatric population. Limited access to services and concern about possible exposure to severe acute respiratory syndrome coronavirus-2 (SARS-CoV-2) resulted in the acceptance of tele-rehabilitation by many patients, providing relief from social isolation and contributing to the medical needs of the elderly [119]. The response to the COVID-19 pandemic shows that lessons were learned from the 2008 crisis. However, budgetary pressures are likely to increase in the coming years, so it is imperative that countries act now. A key concern is to fundamentally shift the priorities of health systems towards PHC, posing numerous policy challenges [50].

Reducing the gap by focusing on vulnerable populations is where public health policymakers, doctors, and clinicians need to invest, as well as increasing research studies on inequalities in access to and use of health services in these populations in times of crisis [115]. In older patients with less education and lower income [120] in a study in the United States, an inability to use telemedicine was more often indicated. Another study [121] also indicated that a key barrier to access and use of eHealth, in the older population, is unfamiliarity with online applications [106]. Therefore, we recommend strengthening PHC and access to secondary care, which has been on the rise in the years of austerity and could be achieved through the introduction of mandatory referrals. Health prevention and promotion services remain the cornerstone of a high-quality health system, simultaneously decongesting secondary and tertiary health care and leading to good levels of population health [122]. It is also necessary to strengthen the quality of care through the development of clinical guidelines and quality indicators [23].

Therefore, health policymakers could focus on a capitalist model with more stakeholders, while others focus on a “green recovery” with improved funding for health and social protection [123]. Certainly, addressing the pre-pandemic crises of income inequality and climate change requires progressive tax reforms and innovations in fiscal policy to avoid a new era of austerity. Policies to eliminate barriers to access to health care are essential to ensure health for all [124].

Austerity policies have directly impacted health services in various countries. For example, Greece and Portugal committed to the International Monetary Fund and faced significant reductions in their national health budgets. Additionally, Spain, Italy, Bulgaria, Croatia, Estonia, Hungary, Iceland, Ireland, Latvia, and Romania experienced similar reductions. Moreover, some countries, like Greece, Bulgaria, and Latvia, saw a decline in social security revenues due to rising unemployment. Conversely, Austria, the Czech Republic, and Poland saw both their revenues and expenditure increase despite the economic crisis. In France and Germany, funding for health services increased, while Belgium, Norway, and England were not affected by austerity measures. Evidently, the European health systems had to overcome numerous challenges due to the austerity measures, such as strengthening universal access to health care, improving service quality, addressing workforce aging and shortages, promoting interdisciplinary cooperation, continuing education, and utilizing modern digital media. However, not all countries managed to overcome these challenges, since to do so necessitated the presence of efficient governance, strategic leadership, accountability, and transparent evaluation across all levels of the health care system.

### 4.1. Study Contributions and Implications

The main contribution of this study is the highlighting of health inequalities, especially between southern and northern European countries, as a result of the economic crisis of the 2010s and the need for health policies to invest in increasing universally accessible services for screening and primary health care. Additionally, there is a need for proper recording of health needs, with databases in all countries, in order to monitor and compare data properly, with the aim of developing health policies with a long-term horizon. Furthermore, it emphasizes the significant impact of measures to tackle economic hardship on health service deprivation and underscores the role of the welfare state in ensuring equal and universal access to health services.

The pandemic has caused disruption to the normal use of health services, where, following the period of economic crisis in Europe, new approaches are needed to meet the universal health needs of the population. Governments need to improve the medical environment and primary health care services provided, reducing the gap with hospital care and emphasizing public education and health promotion. At the same time, the use of telemedicine can improve accessibility, especially in areas with low levels of access to health services. Challenges for the effective and efficient implementation of telemedicine include adapting the elderly population to the use of new technology, ensuring reliable Internet connections, and evaluating the results of telehealth interventions. Attention is also needed to address issues of unmet health needs during the pandemic.

The key health policy strategies, reflected in the action plans of European countries, include bridging primary health care provision with public health actions to improve health and well-being throughout the life course, prevention and control of non-communicable chronic diseases, universal access, measures to tackle antimicrobial resistance and infections, vaccine management and coverage, rapid response to emergencies, prevention and control of non-communicable diseases, prevention and control of chronic diseases, vaccine management and vaccination coverage, and the development of a comprehensive health care system.

In line with the 2024 European Council conclusions on the EU strategy for global health and the promotion of equitable access to health services and commodities, there is a need to collectively strengthen health financing at global, regional, and national levels, mobilize domestic resources in partner countries, and to report regularly on the results of the joint strategy.

### 4.2. Limitations

Caveats are mentioned for the comparison of results between 2004/5 and 2019/20 as there is a difference in the methodology for constructing the health service use indicators and the composition of the populations. Many components of both indicators and accessibility have changed between Waves 1 and 8 (not collected or replaced by other parameters). However, a significant effort was made to ensure that the components in the indicators are indicative and, as far as possible, reflect both secular trends and within Wave 8 comparisons with geographic areas; their characteristics are also compared in terms of accessibility for all populations (50+ years old).

It is important to mention that during the study, the central management team incorporated new scientific tools or enhanced existing ones (such as incorporating new questions) between waves. These modifications could account for some slight inconsistencies when comparing data across waves. However, the required weights have been utilized as advised by the central management team, and despite the changes between waves, the research objectives have remained unchanged.

## 5. Conclusions

Our findings suggest that the economic crisis was associated with a decrease in the use of health services. In the 2010s, a low use of preventive health services was recorded, mainly in southern European countries. Lack of accessibility/availability of health care services (LAAHCS) showed a significant increase from 2004/5 to 2019/20, and central European countries showed higher average PHSU and HCSU scores than the corresponding other countries in Europe. This lack was associated with lower prevention and health service use scores. Therefore, it is necessary to design and implement health policies in all European countries, so as to address the imminent increase in health service needs, given the increase in the elderly population.

## Figures and Tables

**Table 1:** Characteristics of 46,106 Europeans aged 50+ participating in Survey of Health, Ageing and Retirement in Europe (SHARE) (8th Wave, 2019/20).

		n	%	Mean ± Stand. Dev.
**European region**	*North*	10,839	23.5	
	*Central*	23,758	51.5	
	*South*	11,509	25.0	
**Gender**	*♂*	19,641	42.6	
	*♀*	26,465	57.4	
**Age**, *years*	*50–59*	4696	10.2	71.3 ± 9.3
	*60–69*	16,208	35.2	
	*70–79*	15,601	33.8	
	*80–89*	8165	17.7	
	*90–104*	1436	3.1	
**Education**, *years*	*0*	1549	3.4	11.8 ± 4.5
	*1–7*	6722	14.6	
	*8–12*	24,766	53.7	
	*13+*	13,069	28.3	
**Family status**	*Unmarried*, *divorced*, *widow*	5912	12.8	
	*Married*, *living with partner*	40,194	87.2	
**Occupation**	*Employed*	9619	20.9	
	*Unemployed*, *retired*, *housemaker*	36,487	79.1	
**Chronic conditions or diseases**	*None*	11,922	25.9	
	*1*	12,890	28.0	
	*2*	10,085	21.9	
	*3+*	11,209	24.2	

**Table 2:** Seven components of preventive health services utilization score (PHSUs) in 46,106 European adults, aged 50+ years.

Seven PHSUs Components	*Relevant Questions*	n	Estimated Population	
			N	Weight % (95% CIs)
Supplementary Insurance	*Long-term care insurances: private voluntary/supplementary*	3309	13,884,880	7.7 (7.0, 8.4)
Having flu vaccination	*In the last year, have you had a flu vaccination?*	16,205	58,635,846	32.4 (31.4, 33.4)
Having eye examination	*In the last two years, have you had an eye exam performed by an eye care professional such as an ophthalmologist or optometrist?*	23,504	91,418,130	50.5 (49.4, 51.7)
Having a mammogram	*If you are a woman: In the last two years, have you had a mammogram (X-ray of the breast)?*	11,554	46,075,682	25.5 (24.5, 26.5)
Having a Colon Cancer Screening	*Some health care providers do tests such as test for detecting hidden blood in your stool, sigmoidoscopy or colonoscopy to check for colon cancer. In the past two years, have you had any of these tests?*	13,095	51,735,025	28.6 (27.5, 29.7)
Having a planned Hospitalization	*How many times have you been a patient in a hospital overnight during the last twelve months? Was this stay in hospital planned or was it an emergency?*	2193	8,664,028	4.8 (4.4, 5.2)
Polypharmacy	*Do you take at least five different drugs on a typical day? Please include drugs prescribed by your doctor, drugs you buy without prescription, and dietary supplements such as vitamins and minerals.*	11,785	39,843,235	22.0 (21.2, 22.9)

95% CIs: 95% confidence intervals.

**Table 3:** Fifteen components of health care services utilization score (HCSUs) in 46,106 European adults, aged 50+ years.

Fifteen HCSUs Components	*Relevant Questions*	*Scoring*	n	Estimated Population	
				N	Weight % (95% CIs)
Times talked to medical doctor/nurse	*During the last 12 months, about how many times in total have you seen or talked to a medical doctor or qualified/registered nurse about your health? Please exclude dentist visits and hospital stays, but include emergency room or outpatient clinic visits.*	*0: 0 times*	4817	17,998,105	9.9 (9.3, 10.6)
		*1: 1–2*	9464	34,655,741	19.2 (18.2, 20.1)
		*2: 3–6*	16,652	66,477,847	36.8 (35.6, 37.9)
		*3: 7–12*	9742	39,509,227	21.8 (20.9, 22.8)
		*4: 13+*	5431	22,246,043	12.3 (11.6, 13.0)
Contact with general practitioners	*How many of these contacts were with a general practitioner or with a doctor at your health care center?*	*0: 0*	7717	28,637,309	15.8 (14.9, 16.8)
		*1: 1–2*	15,164	54,939,052	30.4 (29.3, 31.5)
		*2: 3–6*	16,495	67,761,589	37.5 (36.3, 38.6)
		*3: 7–12*	5459	23,494,351	13.0 (12.4, 13.6)
		*4: 13+*	1271	6,054,661	3.3 (3.0, 3.8)
Contact with specialists	*How many of these contacts were with a specialist, excluding dentist and emergency visits?*	*0: 0*	18,041	68,134,063	37.7 (36.5, 38.8)
		*1: 1–2*	13,916	56,704,926	31.3 (30.3, 32.5)
		*2: 3–6*	10,106	40,344,497	22.3 (21.3, 23.3)
		*3: 7–12*	2816	10,579,722	5.8 (5.4, 6.3)
		*4: 13+*	1227	5,123,754	2.8 (2.5, 3.3)
Patient in hospital	*How many times have you been a patient in a hospital overnight during the last twelve months?*	*0: 0 times*	38,819	153,869,471	85.1 (84.3, 85.8)
		*1: 1*	4625	17,297,478	9.6 (8.9, 10.2)
		*2: 2+*	2662	9,720,013	5.4 (5.0, 5.8)
Patient in a nursing home	*During the last twelve months, have you been in a nursing home/residential care facility overnight? During the last 12 months, how many weeks altogether did you stay in a nursing home or residential care facility?*	*0: 5 or no*	45,933	180,279,344	99.6 (99.6, 99.7)
		*1: yes temporarily or up to 25 weeks*	135	495,644	0.3 (0.2, 0.4)
		*2: yes or 26+ weeks*	38	111,974	0.1 (0.0, 0.1)
Received home care	*received professional home care: nursing or personal care or weeks received nursing care*	*0: not selected*	44,848	176,469,611	97.6 (97.3, 97.8)
		*1: selected or up to 25 weeks*	511	1,681,987	0.9 (0.8, 1.1)
		*2: selected or 26+ weeks*	747	2,735,365	1.5 (1.3, 1.7)
	*received professional home care: domestic tasks or weeks received help from paid professionals*	*0: not selected*	42,755	168,396,146	93.1 (92.7, 93.5)
		*1: selected or up to 25 weeks*	1528	5,159,311	2.8 (2.6, 3.1)
		*2: selected or 26+ weeks*	1823	7,331,505	4.1 (3.7, 4.4)
	*received professional home care: meals-on-wheels or weeks received meals-on-wheels*	*0: not selected*	45,205	178,686,104	98.8 (98.6, 98.9)
		*1: selected or up to 25 weeks*	371	868,904	0.5 (0.4, 0.6)
		*2: selected or 26+ weeks*	530	1,331,954	0.7 (0.6, 0.9)
	*received care from private providers type of received care from private providers*	*0: no*	45,155	176,977,126	97.8 (97.6, 98.1)
		*1: yes*	951	3,909,836	2.2 (1.9, 2.4)
Seeing a dentist/dental hygienist	*During the last twelve months, have you seen a dentist or a dental hygienist?*	*0: no*	20,720	80,788521	44.7 (43.5, 45.8)
		*1: yes*	25,386	100,098,442	55.3 (54.2, 56.5)
Total nights stayed in hospital	*How many nights altogether have you spent in hospitals during the last twelve months?*	*0: 0*	38,847	153,963,575	85.1 (84.3, 85.9)
		*1: 1–2*	1888	6,735,412	3.7 (3.3, 4.2)
		*2: 3–6*	2135	7,773,854	4.3 (4.0, 4.6)
		*3: 7–12*	1616	6,108,481	3.4 (3.0, 3.8)
		*4: 13+*	1620	6,305,639	3.5 (3.1, 3.9)
Stayed overnight in hospital	*During the last twelve months, have you been in a hospital overnight? Please consider stays in medical, surgical, psychiatric or in any other specialised wards.*	*0: no*	38,759	153,733,715	85.0 (84.2, 85.7)
		*1: yes*	7347	27,153,247	15.0 (14.3, 15.8)
Paid for nursing care	*Did you pay anything yourself for nursing home stays or stays in residential care facilities in the last twelve months?*	*0: no*	46,002	180,500,230	99.8 (99.7, 99.8)
		*1: yes*	104	386,732	0.2 (0.2, 0.3)
Hours received professional nursing care	*On average, how many hours per week did you receive professional or paid help with personal care at home?*	*0: 0*	44,949	176,818,131	97.8 (97.5, 98.0)
		*1: 1–2*	328	986,990	0.5 (0.5, 0.7)
		*2: 3–6*	266	955,870	0.5 (0.4, 0.6)
		*3: 7–12*	199	909,094	0.5 (0.4, 0.6)
		*4: 13+*	364	1,216,878	0.7 (0.6, 0.8)
Hours received paid domestic help	*On average, how many hours per week did you receive such professional or paid help?*	*0: 0*	42,940	168,989,395	93.4 (93.0, 93.8)
		*1: 1–2*	1211	4,459,210	2.5 (2.2, 2.7)
		*2: 3–6*	1169	4,580,602	2.5 (2.3, 2.8)
		*3: 7–12*	358	1,421,929	0.8 (0.7, 0.9)
		*4: 13+*	428	1,435,827	0.8 (0.7, 0.9)

**Table 4:** Sixteen components of lack of accessibility/availability in health care services (LAAHCS) in the sample of 46,105 European adults, aged 50+ years.

Components	*Relevant Questions*	n	Estimated Population	
			N	Weight % (95% CIs)
*Forgo care due to cost*	*General practitioner*	397	2,197,002	1.20 (1.00, 1.50)
	*Specialist physician*	1031	4,598,815	2.50 (2.20, 3.00)
	*Drugs*	635	2,409,460	1.30 (1.20, 1.50)
	*Dental care*	1959	8,186,107	4.50 (4.00, 5.10)
	*Optical care*	735	3,099,292	1.70 (1.50, 2.00)
	*Home care*	273	1,087,645	0.60 (0.50, 0.70)
	*Paid home help*	483	1,803,858	1.00 (0.90, 1.20)
	*Other*	427	2,047,961	1.10 (0.90, 1.40)
*Forgo care due to unavailability*	*General practitioner*	408	1,919,744	1.10 (0.90, 1.30)
	*Specialist physician*	1241	5,311,113	2.90 (2.50, 3.40)
	*Drugs*	277	1,081,254	0.60 (0.50, 0.80)
	*Dental care*	697	2,995,690	1.70 (1.40, 2.00)
	*Optical care*	509	2,151,853	1.20 (1.00, 1.40)
	*Home care*	175	517,723	0.30 (0.20, 0.40)
	*Paid home help*	197	787,022	0.40 (0.30, 0.60)
	*Other*	369	1,815,148	1.00 (0.80, 1.30)

95% CIs: 95% confidence intervals.

**Table 5:** Score levels of preventive health services utilization (PHSU) and health care services utilization (HCSU) in the sample of 46,106 European adults, aged 50+ years, between European regions.

European Region	n	Estimated Population	Preventive Health Services Utilization Score (PHSUs) ^a^		Health Care Services Utilization Score (HCSUs) ^a^		Δ-Difference	
			Weighted Mean	95% CI	Weighted Mean	95% CI	Weighted Mean	95% CI
**North**	10,839	10,897,179	26.2	25.7, 26.8	13.4	13.1, 13.8	12.8	12.2, 13.3
**Central**	23,758	113,398,726	25.5	25.1, 25.9	17.5	17.2, 17.7	8.0	7.6, 8.4
**South**	11,509	56,591,058	22.2	21.4, 23.0	15.8	15.3, 16.3	6.4	5.6, 7.2

PHSUs: preventive health services utilization score; HCSUs: health care services utilization score; 95% CI: 95% confidence interval. ^a^ Scores range from 0 to 100, with a higher score indicating greater use of health services. The overall mean PHSU score was 24.6 (95% CI: 24.3, 25.0), HCSU score was 15.5 (95% CI: 15.4, 15.8), and Δ-difference was 9.1 (95% CI: 8.7, 9.4). Estimations were based on complex samples analysis (ANCOVA: gender, age, education, family status, occupation, and chronic conditions or diseases were used as covariates).

**Table 6:** Score levels of preventive health services utilization (PHSU) and health care services utilization (HCSU) in the sample of 46,105 European adults, aged 50+ years, according to score frequency of lack of accessibility/availability in health care services (LAAHCS).

		Preventive Health Services Utilization Score (PHSUs) ^a^		Health Care Services Utilization Score (HCSUs) ^a^		Δ-Difference	
		Weighted Mean	SE	Weighted Mean	SE	Weighted Mean	SE
**Score Frequency of Lack of Accessibility/Availability in Health Care Services ^b^**	*0 (none)*	24.71	0.19	16.64	0.12	8.07	0.18
	*1 to 24 (partial)*	23.52	0.53	17.47	0.35	6.05	0.50
	*25+ (high)*	18.52	1.18	14.61	0.86	3.91	1.01
	*p*-value	<0.001		0.004		<0.001	
	*p*-trend	0.001		0.019		<0.001	

SE, standard error of mean. ^a^ Scores range from 0 to 100, with a higher score indicating greater use of health services (^b^ or lack of accessibility/availability in health care services). Comparisons across score frequency were based on complex samples analysis (ANCOVA: gender, age, education, family status, occupation, chronic conditions or diseases, and European regions were used as covariates).

## Data Availability

The data that support the findings of this study are available from the corresponding author upon reasonable request.

## Supplementary Materials

The following supporting information can be downloaded at: https://www.mdpi.com/article/10.3390/healthcare12090928/s1, Table S1: Score frequency of lack of accessibility/availability in health care services (LAAHCS) in relation to characteristics of 46,105 European adults aged 50+ years.
