# Supplementary material for: Utilization of Health Care Services and Accessibility Challenges among Adults Aged 50+ before and after Austerity Measures across 27 European Countries: Secular Trends in the SHARE Study from 2004/05 to 2019/20
Source: Healthcare (Basel). 2024 Apr 30;12(9):928. doi: 10.3390/healthcare12090928 (PMC11083176; doi:10.3390/healthcare12090928)
Supplement: Supplementary file 1 [file healthcare-12-00928-s001.zip › healthcare-2942381-supplementary.pdf]

Supplementary Materials

Table S1. Score frequency of lack of Accessibility - Availability in Health Care Services (LAAHCS) in relation to characteristics of 46,105 Europeans adults aged 50+ years.

			Score frequency of lack of Accessibility - Availability in Health Care Services [a]			
			**0**	**1 to 24**	**25+**	
			n=40,728	n=4874	n=503	
		n	weighted %			p-value
**Gender**	♂	19,641	88.8	10.3	0.9	<0.001
	♀	26,464	85.5	13.3	1.1	
**Age,** *years*	*50-59*	4696	85.1	14.2	0.7	0.088
	60-69	16,208	87.4	11.6	1.0	
	70-79	15,601	88.2	10.6	1.2	
	80-89	8164	87.5	11.4	1.1	
	90-104	1436	84.6	14.3	1.1	
**Education,** *years*	*0*	1549	83.5	15.4	1.1	0.015
	1-7	6721	84.4	14.1	1.4	
	8-12	24,766	87.4	11.5	1.1	
	13+	13,069	88.5	11.0	0.5	
**Family status**	*unmarried, divorced, widow*	5911	83.5	14.2	2.3	<0.001
	married, living with partner	40,194	87.6	11.6	0.8	
**Occupation**	*employed*	9619	87.1	12.3	0.6	0.017
	unemployed, retired, housemaker	36,486	87.0	11.8	1.2	
**Chronic conditions or diseases**	*none*	11,921	89.4	10.0	0.6	<0.001
	1	12,890	87.0	11.8	1.2	
	2	10,085	87.2	12.0	0.8	
	3+	11,209	83.5	14.9	1.6	

[a] Score range from 0 to 100, with a higher score indicating higher lack of Accessibility - Availability in Health Care Services.

χ^2 tests.
